# Multifaceted interactions and regulation between antizyme and its interacting proteins cyclin D1, ornithine decarboxylase and antizyme inhibitor

**DOI:** 10.18632/oncotarget.4469

**Published:** 2015-06-26

**Authors:** Yen-Chin Liu, Chien-Yun Lee, Chi-Li Lin, Hui-Yi Chen, Guang-Yaw Liu, Hui-Chih Hung

**Affiliations:** ^1^ Department of Life Sciences, National Chung Hsing University (NCHU), Taichung, Taiwan; ^2^ Graduate Institute of Biotechnology, National Chung-Hsing University (NCHU), Taichung, Taiwan; ^3^ Molecular and Biological Agricultural Sciences Program, Taiwan International Graduate Program, Academia Sinica, Taipei, Taiwan; ^4^ Institute of Medicine, Chung Shan Medical University, Taichung, Taiwan; ^5^ Biotechnology Center, National Chung-Hsing University (NCHU), Taichung, Taiwan; ^6^ Agricultural Biotechnology Center (ABC), National Chung-Hsing University (NCHU), Taichung, Taiwan; ^7^ Institute of Microbiology & Immunology, Chung Shan Medical University, Taichung, Taiwan; ^8^ Division of Allergy, Immunology, and Rheumatology, Chung Shan Medical University Hospital, Taichung, Taiwan; ^9^ Institute of Genomics and Bioinformatics, National Chung Hsing University (NCHU), Taichung, Taiwan

**Keywords:** biochemistry, molecular and cellular biology, signal transduction, cell cycle, oncogene

## Abstract

Ornithine decarboxylase (ODC), cyclin D1 (CCND1) and antizyme inhibitor (AZI) promote cell growth. ODC and CCND1 can be degraded through antizyme (AZ)-mediated 26S proteasomal degradation. This paper describes a mechanistic study of the molecular interactions between AZ and its interacting proteins. The dissociation constant (*K*_d_) of the binary AZ-CCND1 complex and the respective binding sites of AZ and CCND1 were determined. Our data indicate that CCND1 has a 4-fold lower binding affinity for AZ than does ODC and an approximately 40-fold lower binding affinity for AZ than does AZI. The *K*_d_ values of AZ-CCND1, AZ-ODC and AZ-AZI were 0.81, 0.21 and 0.02 μM, respectively. Furthermore, the *K*_d_ values for CCND1 binding to the AZ N-terminal peptide (AZ_34–124_) and AZ C-terminal peptide (AZ_100–228_) were 0.92 and 8.97 μM, respectively, indicating that the binding site of CCND1 may reside at the N-terminus of AZ, rather than the C-terminus. Our data also show that the ODC-AZ-CCND1 ternary complex may exist in equilibrium. The *K*_d_ values of the [AZ-CCND1]-ODC and [AZ-ODC]-CCND1 complexes were 1.26 and 4.93 μM, respectively. This is the first paper to report the reciprocal regulation of CCND1 and ODC through AZ-dependent 26S proteasomal degradation.

## INTRODUCTION

Cyclin D1 (CCND1) serves as an active switch in the regulation of the G1-to-S phase transition during cell cycle progression [1], specifically functioning as an allosteric regulator of cyclin-dependent kinase 4 (CDK4) [1, 2]. CCND1 binds to and activates CDK4 to form an active complex that promotes cell cycle progression by phosphorylating and inactivating the retinoblastoma protein (Rb) [3–5], which is essential for the activation of gene expression networks that regulate entry into and progression through the S phase. Recent studies have shown that CCND1 also exhibits CDK4-independent activity, functioning as a transcriptional coregulator by directly binding to transcription factors and regulating the activity of histone acetylase and deacetylase [1, 6]. Because CCND1 is an important regulator of cell cycle progression and can function as a transcriptional coregulator, overexpression of CCND1 and deregulation of CCND1 degradation are thought to be associated with the development and progression of cancer [7–11 and references therein]. Therefore, CCND1 is considered as an attractive target for anti-cancer therapy, with several agents currently in development [12–18].

Multiple pathways are involved in the turnover of CCND1 [9, 18, 19], which is mediated by glycogen synthase kinase 3β (GSK3β) or by p38 phosphorylation, ubiquitination and binding to antizyme (AZ) [18, 19]. The CCND1 protein is primarily degraded through the ubiquitin-dependent 26S proteasomal degradation pathway [20, 21], which involves GSK3β-, p38^SAPK2^- or ERK2-mediated phosphorylation of CCND1 at Thr 286 [21–26]. In addition, phosphorylation at Thr 288 by the Mirk/Dyrk 1b kinase has been shown to regulate CCND1 stability [27]. A ubiquitin-independent pathway mediated by AZ has also been observed to be involved in the degradation of CCND1 [19]. Thus, in addition to ubiquitin-dependent proteasomal degradation, CCND1 can be regulated by AZ through a ubiquitin-independent pathway, and AZ may play a vital role in regulating cellular levels of CCND1 [19].

AZ is a protein inhibitor of ornithine decarboxylase (ODC), which is the first enzyme and a rate-limiting enzyme in the biosynthesis of polyamines [28–31]. AZ was originally identified as a negative modulator of ODC due to its role in facilitating the degradation of ODC [30, 32, 33]. ODC specifically undergoes a unique type of ubiquitin-independent proteasomal degradation via direct interaction with AZ: the binding of AZ promotes the dissociation of ODC homodimers and targets ODC for degradation by the 26S proteasome [34–38]. In addition, AZ was the first protein that was found to utilize translational frame shifting in the regulation of mammalian mRNA [28, 39]. In particular, increased concentrations of polyamines cause the ribosome to bypass the first open reading frame (ORF) of AZ, allowing a fully functional 22-kDa AZ protein to be synthesized from the second ORF (+1 frame-shift) [39, 40]. AZ is regarded as a tumor suppressor that suppresses cancer cell proliferation and transformation by inhibiting ODC activity and polyamine transport, and it impedes the progression of many cancers that are caused by anomalous ODC and polyamine levels [28, 30, 41–43]. However, the degradation of AZ itself is ubiquitin dependent, and polyamines interfere with AZ degradation [44, 45].

Antizyme inhibitor (AZI) is a negative modulator of AZ that functions by binding to AZ. AZI rescues ODC enzymatic activity, ultimately increasing polyamine levels within the cell. However, although the AZI protein is homologous to ODC, it lacks the enzymatic activity of ODC [46–48]. Because it binds to AZ with a higher affinity than does ODC, AZI can sequester AZ from the AZ-ODC heterodimer and rescue ODC activity from AZ suppression, thereby preventing the rapid degradation of ODC [48–50]. An elevated AZI level increases cellular polyamine concentrations, thus resulting in cell proliferation and transformation [51, 52], suggesting that AZI is also an oncogenic protein [48]. As the ratio of [AZI]/[AZ] within the cell can directly influence tumor growth, it is an important index of the regulation of human cancer [53]. Recent sequencing of the hepatocellular carcinoma (HCC) transcriptome revealed that AZI was modified at the RNA level, resulting in a protein with a serine-to-glycine mutation at residue 367 that shows a stronger affinity toward AZ. This mutant AZI is capable of promoting cell proliferation via neutralization of AZ-mediated ODC and CCND1 degradation [54].

In addition to ODC and CCND1, AZ has been shown to bind to and facilitate the ubiquitin-independent degradation of other cell cycle-regulating proteins, including Aurora-A kinase and Smad1 [55–57]. Aurora-A kinase plays an essential role in mitotic events, and AZ regulates Aurora-A kinase stability through the negative regulator Aurora-A kinase interacting protein 1, which promotes the ubiquitin-independent degradation of Aurora-A kinase. AZ, Aurora-A kinase and Aurora-A kinase interacting protein 1 have been found to exist as a ternary complex [56].

Based on the results described above, the roles of AZ in tumor suppression have been established. First, AZ inhibits the oncogenic enzyme ODC by binding to it directly, forming an ODC-AZ heterodimer and promoting the degradation of ODC by the 26S proteasome. Second, AZ is regulated by AZI, which binds to AZ to sequester it from the AZ-ODC heterodimer, thereby restoring ODC activity. Third, AZ regulates Aurora-A kinase by forming a ternary complex with Aurora-A kinase interacting protein 1. Fourth, AZ may play a crucial role in regulating cellular CCND1 levels through a ubiquitin-independent pathway by binding to CCND1, thereby promoting the degradation of CCND1 by the 26S proteasome.

CCND1 is a critical regulator of the cell cycle, and ODC plays a critical role in regulating cell growth and transformation. However, it remains to be determined whether AZ binds to ODC with a stronger affinity than it does to CCND1 and how ODC and CCND1 influence AZ-mediated protein degradation. In this study, precise measurement of the dissociation constant (*K*_d_) of the binary AZ-CCND1 complex and determination of the respective binding sites of AZ and CCND1 were achieved via analytical ultracentrifugation. In addition, the molecular interactions between AZ and its interacting proteins, or ODC, CCND1 and AZI, were investigated. We found that the ODC-AZ-CCND1 ternary complex may exist in equilibrium and that the AZ-mediated degradation of ODC and CCND1 is inhibited by ternary complex formation.

## RESULTS

### Binding affinity of the AZ-CCND1 complex

**Figure 1:**
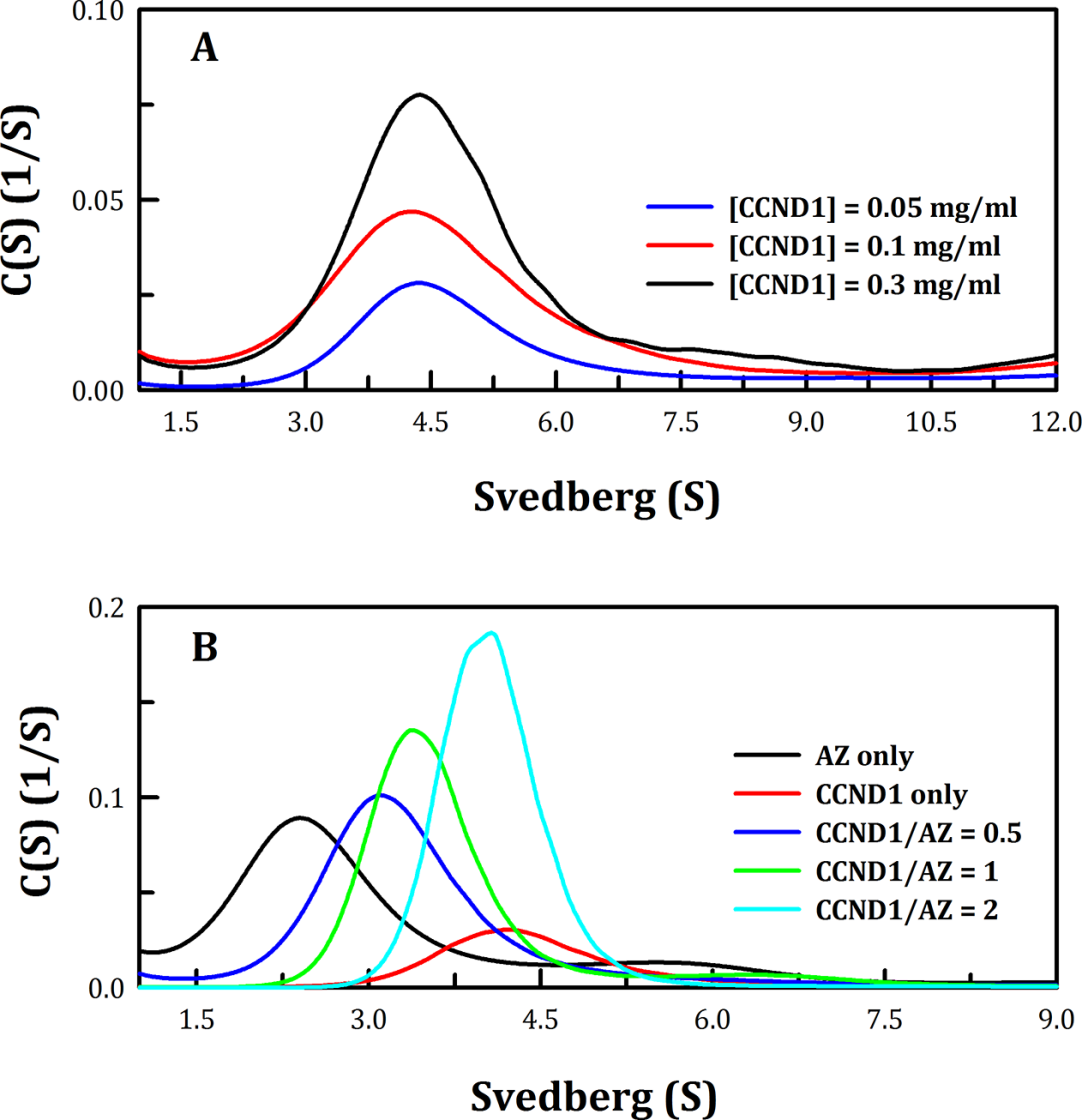
Continuous sedimentation coefficient distribution of the human CCND1 protein and the AZ-CCND1 complex **A.** Three concentrations of the CCND1 protein in 30 mM Tris-HCl buffer (pH 7.4) at 20°C were used in the experiment: 0.05, 0.1 and 0.3 mg/ml. The sedimentation velocity data were globally fitted using the SEDPHAT program to obtain the *K*_d_ of the CCND1 dimer (Table 1). **B.** The concentration of AZ was fixed at 0.25 mg/ml, and CCND1 concentrations of 0.19, 0.37 and 0.74 mg/ml (the molar ratios of CCND1/AZ were 0.5, 1 and 2, respectively) in a buffer containing 30 mM Tris-HCl (pH 7.4) and 50 mM NaCl were used. The sedimentation velocity data were globally fitted using SEDPHAT to obtain the *K*_d_ of the AZ-CCND1 complex (Table 1).

Size distribution analysis demonstrated that the CCND1 protein was present as a dimer with a *K*_d_ of 3.84 μM (Figure 1A and Table 1). This is the first report that the CCND1 protein exists as a dimer in solution and the first description of its *K*_d_ in monomer-dimer equilibrium. Furthermore, size distribution analysis of CCND1 in the presence of AZ indicated that CCND1 bound directly to the AZ protein, forming the AZ-CCND1 complex (Figure 1B). When AZ was not present, CCND1 existed as a dimer, with an S value of approximately 4.2–4.4 (Figure 1A). In contrast, this CCND1 dimer dissociated when AZ was present, producing the AZ-CCND1 heterodimer, and the S value of the heterodimer was approximately 3.4 at a molar ratio of 1 (green line, Figure 1B). Similar to the mode of AZ binding to ODC, AZ bound to CCND1 and induced dissociation of the CCND1 dimer, resulting in formation of the AZ-CCND1 heterodimer (Figure 1B). The *K*_d_ of the AZ_WT_-CCND1 complex, as determined by varying the concentration of CCND1, was 0.81 μM, which was 4-fold higher than that of the AZ_WT_-ODC complex (0.21 μM) and 40-fold higher than that of the AZ_WT_-AZI complex (0.02 μM). These results suggest that among these AZ-interacting proteins, CCND1 binds to AZ with a binding affinity that is weaker than that of ODC and much weaker than that of AZI (Table 1).

**Table 1:** *K*_d_ values of the human AZ-ODC, AZ-AZI and AZ-CCND1 complexes

Protein or protein complex	*K*_d_ (μM)
CCND1 dimer	^a^3.84 ± 0.025
AZ_WT_-ODC	^b^0.21 ± 0.001
AZ_WT_-AZI	^b^0.02 ± 0.009
AZ_WT_-CCND1	^c^0.81 ± 0.02
AZ_34–124_-CCND1	^c^0.87 ± 0.01
AZ_100–228_-CCND1	^c^14.1 ± 0.06
[AZ-ODC]-CCND1	^d^4.93 ± 0.02
[AZ-CCND1]-ODC	^e^1.26 ± 0.02

a The *K*_d_ of the CCND1 dimer was derived from the global fitting of sedimentation velocity data (Figure 1) to the monomer-dimer equilibrium model in the SEDPHAT program.

b These values are derived from a paper published by our group in 2011 [50].

c The *K*_d_ of the AZ-CCND1 complex was derived from the global fitting of sedimentation velocity data (Figures 2 and 4) to the model of A+B↔AB hetero-association in the SEDPHAT program.

d AZ was pre-incubated with ODC for 1 hour to form the AZ-ODC complex, followed by CCND1 addition.

e AZ was pre-incubated with CCND1 for 1 hour to form the AZ-CCND1 complex, followed by ODC addition.

To identify the CCND1-binding domain on the AZ protein, an N-terminal AZ peptide, AZ_34–124_, and a C-terminal AZ peptide, AZ_100–228_, were produced to identify the functional domain essential for CCND1 binding. According to the results of the size distribution analysis, CCND1 appeared to favor binding to the N-terminal AZ peptide (AZ_34–124_) over the C-terminal AZ peptide (AZ_100–228_) (Figure 2A). The protein peak of the AZ_34–124_-CCND1 complex (green line, Figure 2A) fell between that of the AZ_34–124_ protein (red line, Figure 2A) and that of the CCND1 protein (black line, Figure 2A). However, the mixture of AZ_100–228_ and CCND1 did not form protein complexes, as the protein peak of the mixture (cyan line, Figure 2A) was not significantly shifted and was close to that of the AZ_100–228_ protein (blue line, Figure 3A).

**Figure 2:**
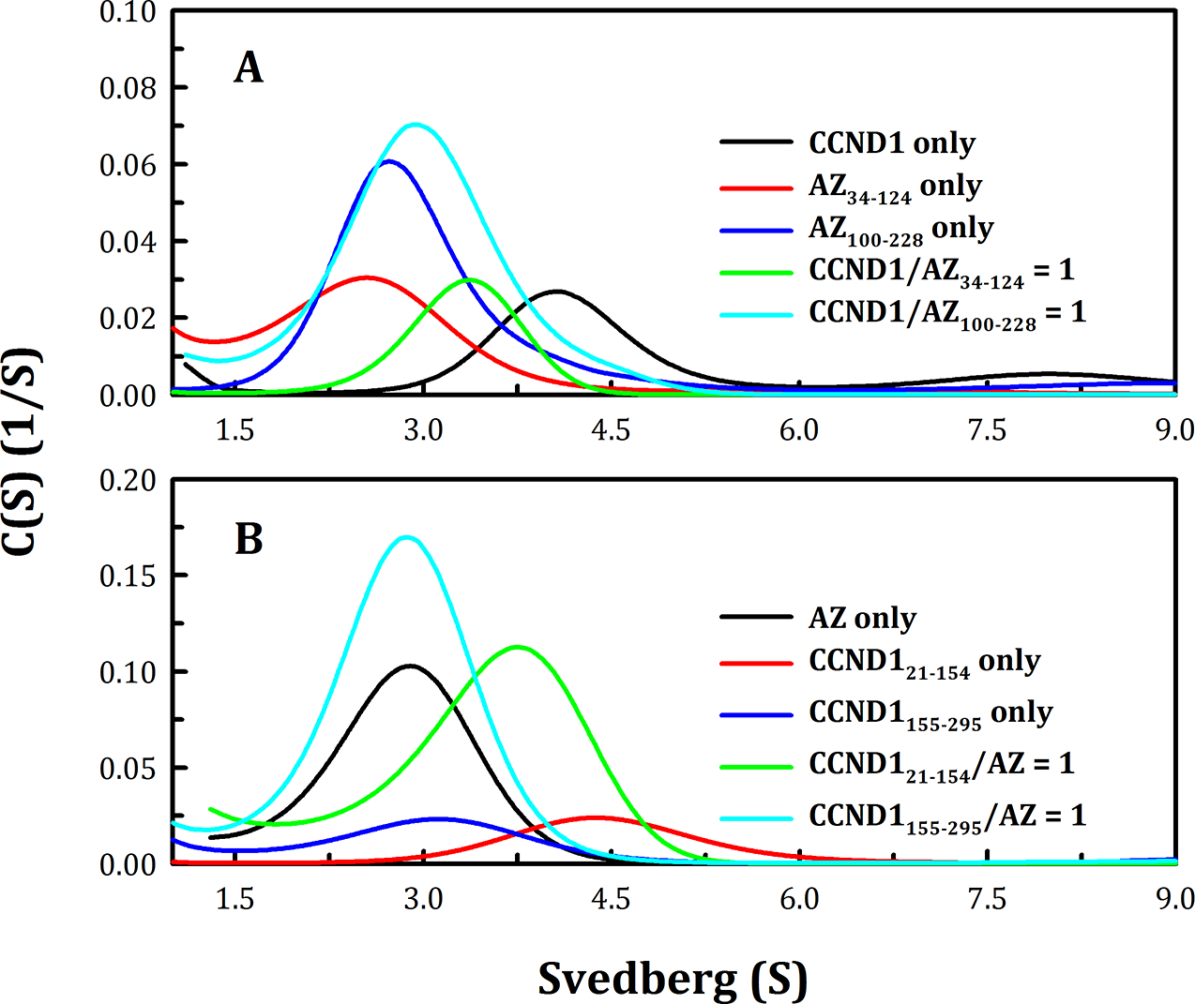
Interactions between the N-terminal and the C-terminal domains of human AZ and CCND1 The protein concentrations of AZ, AZ_34–124_, AZ_100–228_, CCND1, CCND1_21–154_ and CCND1_155–295_ were 0.25, 0.075, 0.077, 0.17, 0.4 and 0.35 mg/ml, respectively, in a buffer containing 30 mM Tris-HCl (pH 7.4) and 50 mM NaCl. The sedimentation velocity was determined at 20°C, and the molar ratio of AZ/CCND1 was fixed at 1.

**Figure 3:**
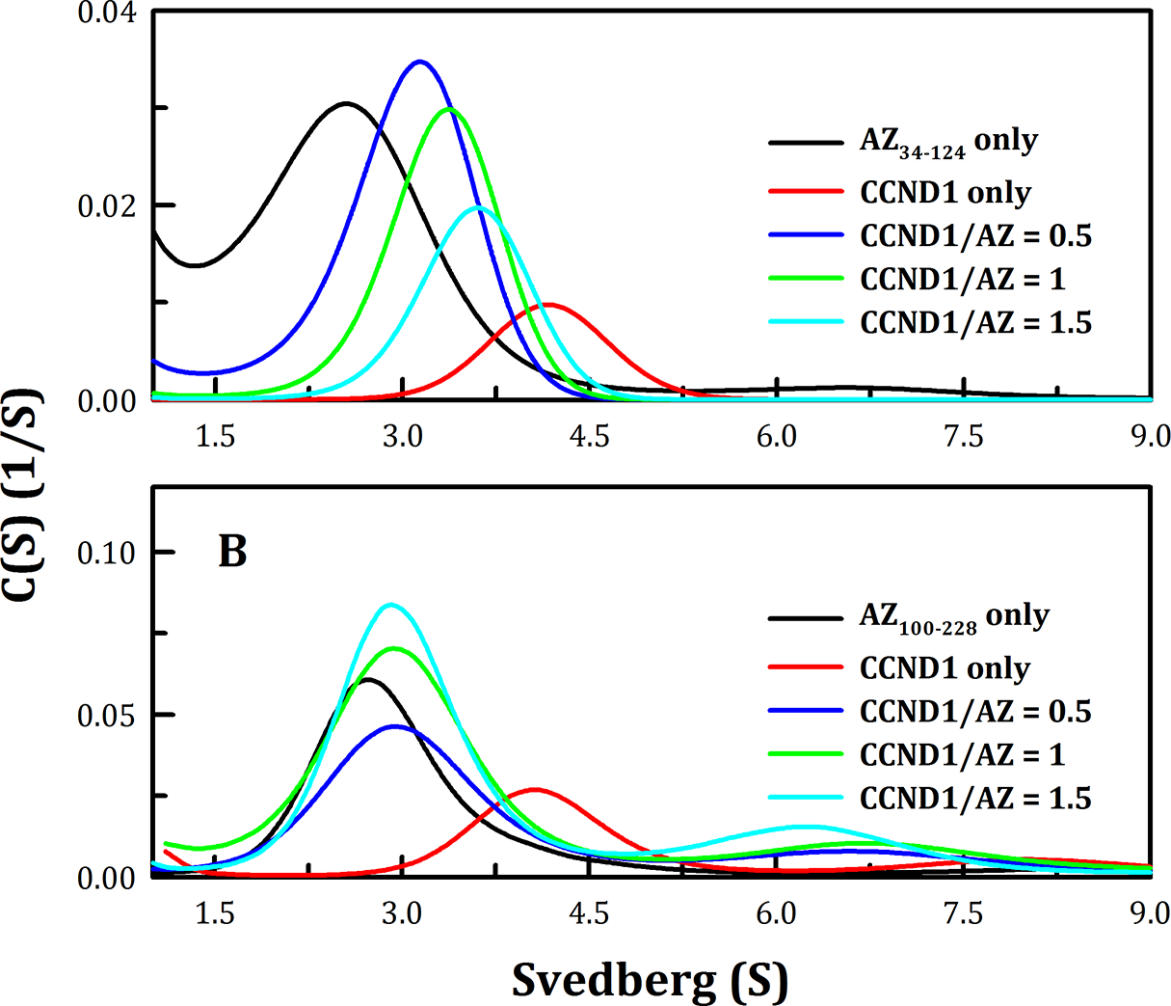
Continuous sedimentation coefficient distribution of human CCND1 in the presence of AZ_34–124_ and AZ_100–228_ The concentrations of AZ_34–124_ and AZ_100–228_ were fixed at 0.1 and 0.25 mg/ml, respectively, whereas CCND1 concentrations of 0.12, 0.25 and 0.37 mg/ml for AZ_34–124_ and of 0.17, 0.35 and 0.52 mg/ml for AZ_100–228_ (corresponding to molar ratios of CCND1/AZ of 0.5, 1 and 1.5, respectively) in a buffer containing 30 mM Tris-HCl (pH 7.4) and 50 mM NaCl were used. The sedimentation velocity data were globally fitted using SEDPHAT to obtain the *K*_d_ of the AZ-CCND1 complex (Table 1).

An N-terminal CCND1 peptide, CCND1_21–154_, and a C-terminal CCND1 peptide, CCND1_155–295_, were also generated to identify the AZ-binding domain, and the size distribution analysis of the truncated CCND1 proteins indicated that CCND1_21–154_ preferentially bound to AZ over CCND1_155–295_ (Figure 2B). The protein peak of the AZ-CCND1_21–154_ complex (green line, Figure 2B) fell between that of AZ (black line, Figure 2B) and that of CCND1_21–154_ (red line, Figure 2B). Mixtures of AZ and CCND1_155–295_ did not form protein complexes, as the protein peak was not shifted (cyan line, Figure 2B). Both the CCND1 N-terminal and C-terminal truncated proteins produced a broader peak with a larger S value than expected (Figure 2B); this may have been due to the instability of the truncated CCND1 proteins, which tended to polymerize.

The *K*_d_ values of the AZ_34–124_-CCND1 and AZ_100–228_-CCND1 complexes were also determined. Figure 3 shows the size distribution plots obtained for these protein complexes at different molar ratios of AZ and CCND1. At increasing CCND1 concentrations, the size distribution plot of AZ_34–124_-CCND1 gradually shifted to the right (shown by the blue, green and then cyan lines in Figure 3A). The *K*_d_ of the AZ_34–124_-CCND1 complex was approximately 0.87 μM, similar to that of AZ_WT_-CCND1 (0.81 μM, Table 1). In contrast, the protein peak of the AZ_100–228_-CCND1 complex was not shifted by increasing the CCND1 concentration and consistently produced an S value of approximately 3.0 (blue, green and cyan lines, Figure 3B). The *K*_d_ of the AZ_100–228_-CCND1 complex was approximately 14.1 μM, which is 16-fold higher than that of the AZ_34–124_-CCND1 complex, suggesting that the N-terminus, rather than the C-terminus, of AZ is the major CCND1-binding domain. It is noteworthy that the ODC- and AZI-binding domains in the AZ protein are mainly located at the C-terminus [58]. Therefore, these data suggest that the binding sites of ODC and CCND1 are separated in the AZ protein.

### Mode of binding between AZ and ODC, AZI and CCND1

**Figure 4:**
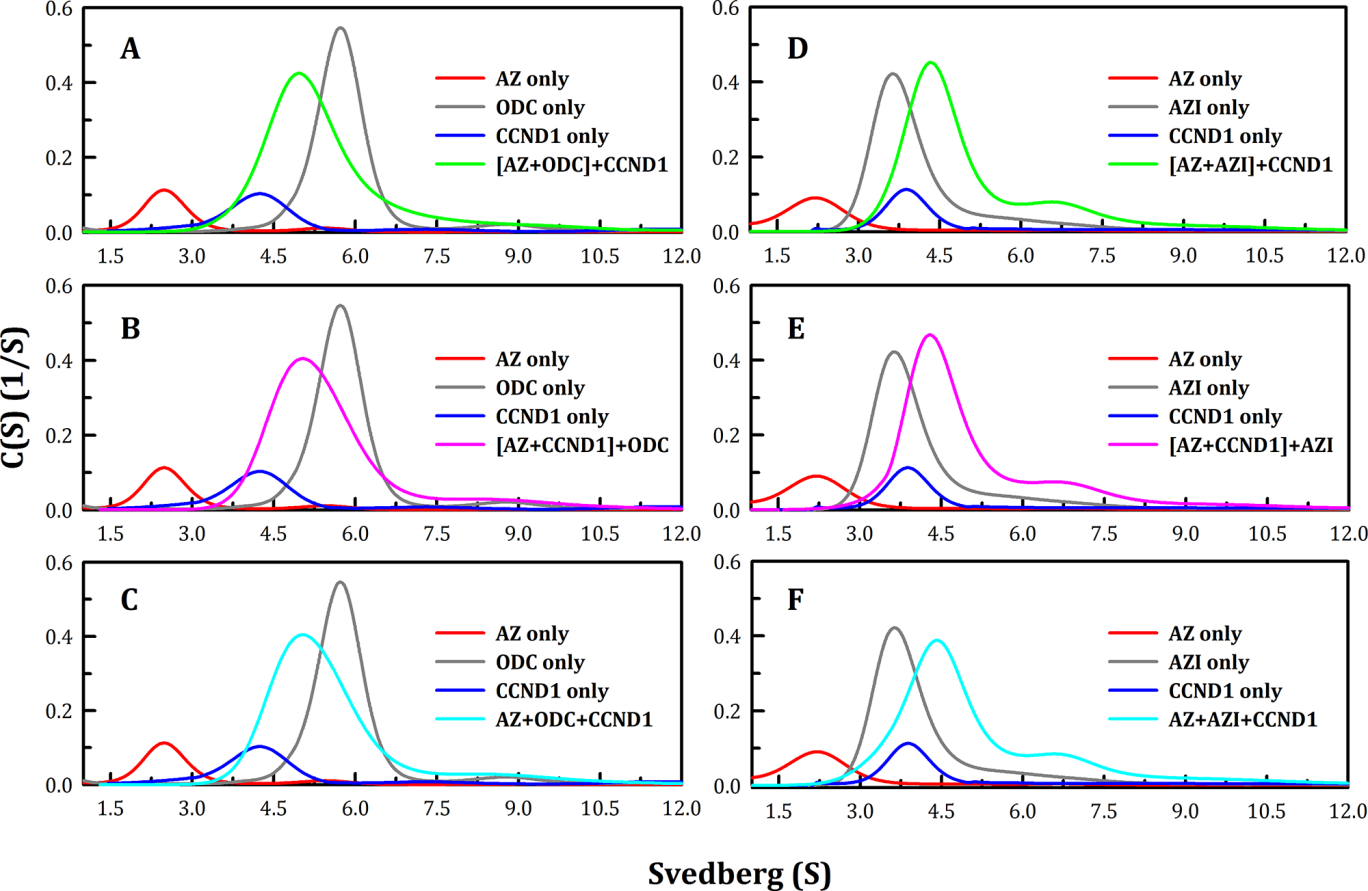
Continuous sedimentation coefficient distribution of AZ with its interacting proteins, ODC, CCND1 and AZI The protein concentrations of AZ, ODC, CCND1 and AZI were 0.2, 0.48, 0.3 and 0.47 mg/ml, respectively, in a buffer containing 30 mM Tris-HCl (pH 7.4) and 50 mM NaCl. The sedimentation velocity was determined at 20°C, and the molar ratio of AZ to each interacting protein was fixed at 1.

**Figure 5:**
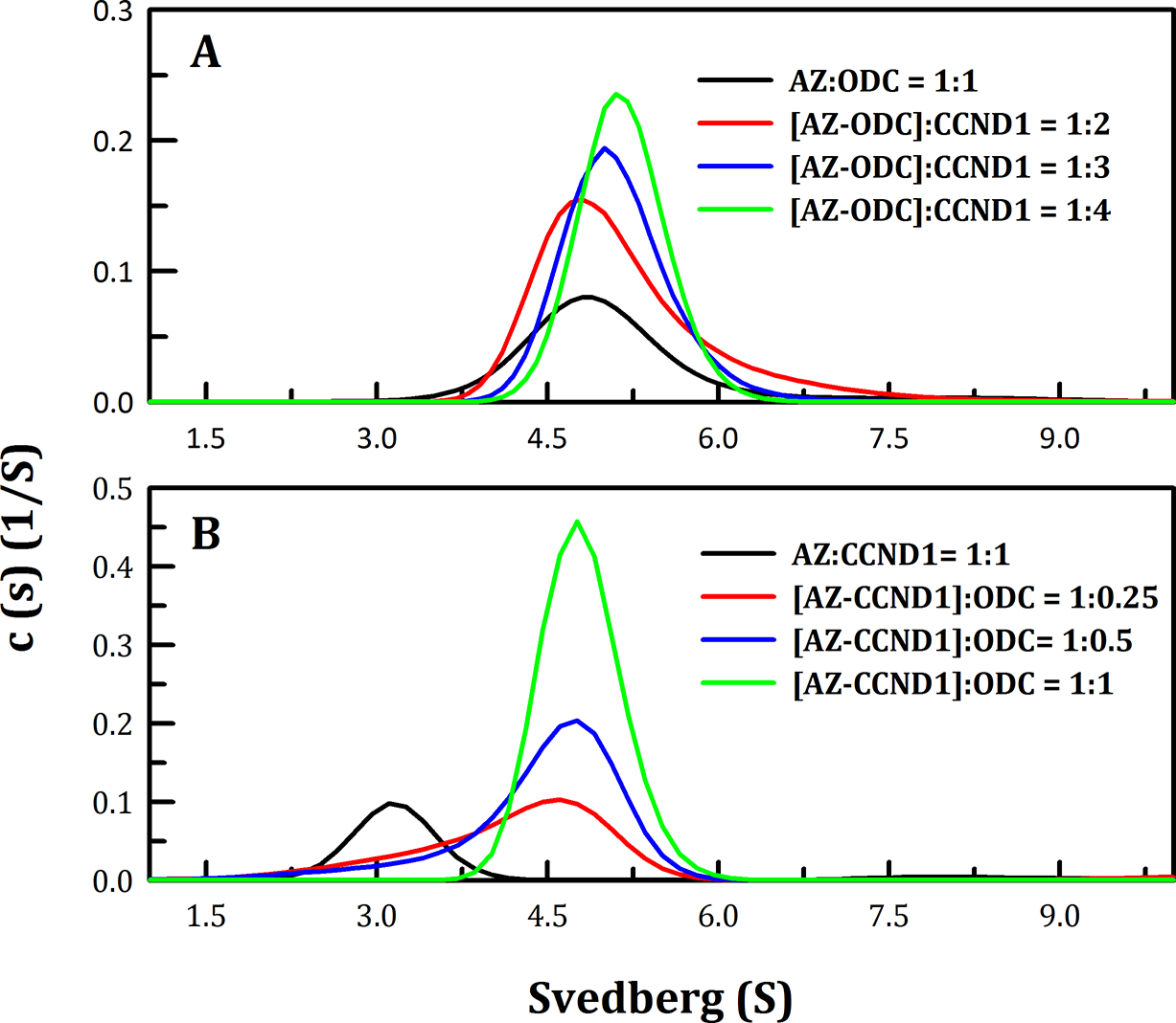
Continuous sedimentation coefficient distributions of the AZ-ODC complex with increasing concentrations of CCND1 and of the AZ-CCND1 complex with increasing concentrations of ODC The sedimentation velocity was determined at 20°C, and the proteins were diluted in a buffer containing 30 mM Tris-HCl (pH 7.4) and 50 mM NaCl. **A.** The molar ratio of AZ/ODC was fixed at 1, and the CCND1 protein concentration was varied. **B.** The molar ratio of AZ/CCND1 was fixed at 1, and the ODC protein concentration was varied.

ODC and AZI bind competitively to AZ at its C-terminus, but with different affinities (50, 58). Hence, we examined complex formation between AZ and its interacting proteins, or ODC, AZI and CCND1. We found that the region to which CCND1 binds on AZ is located in the N-terminal domain of AZ, which is different from the binding regions for ODC and AZI (Figure 3). In addition, we found that the binding affinity of CCND1 for AZ is weaker than that of ODC for AZ (Table 1). Therefore, to examine the mode of AZ binding to these interacting proteins, several sets of sedimentation velocity experiments were designed; the results are illustrated in Figure 4. The first set included pre-incubation of AZ with ODC, followed by treatment with CCND1 ([AZ+ODC]+CCND1, green line, Figure 4A); the second set involved pre-incubation of AZ with CCND1, followed by treatment with ODC ([AZ+CCND1]+ODC, pink line, Figure 4B); and the last set involved co-incubation of AZ, ODC and CCND1 (AZ+ODC+CCND1, cyan line, Figure 4C). However, regardless of the sequence in which these proteins were mixed, the predominant form found under equilibrium conditions was the AZ-ODC protein complex, with an associated S value of approximately 5.0 (green, pink and cyan lines in Figures 4A, and 4C, respectively). Similar results were observed when AZI was substituted for ODC in these experiments. More specifically, when AZ, AZI and CCND1 were all present in equilibrium, the major form observed was the AZ-AZI protein complex, with an S value of approximately 4.3 (green, pink and cyan lines in Figures 4D, and 4F, respectively). These data indicate that AZ preferentially binds to ODC and AZI in the presence of CCND1, even though the binding sites of ODC (or AZI) and CCND1 within AZ are different. However, the ODC-AZ-CCND1 ternary complex may exist in equilibrium, and the S value for this ternary complex is expected to be approximately 6.0. When CCND1 was titrated into the ODC-AZ mixture, the protein peaks gradually shifted toward the right, producing increased S values (red, blue, and green lines, Figure 5A) that were higher than those of the ODC-AZ complex (black line, Figure 5A), thereby indicating that the ODC-AZ-CCND1 ternary complex can form when CCND1 is present in excess. When ODC was titrated into the AZ-CCND1 mixture, the protein peaks significantly shifted toward the right, with increased S values (red, blue, and green lines, Figure 5B) that were higher than those of the AZ-CCND1 complex (black line, Figure 5B) but not higher than those for the ODC-AZ complex (black line, Figure 5A). Therefore, the protein peaks shown in Figure 5B may mostly represent a binary complex in which ODC caused the dissociation of CCND1 from AZ.

The *K*_d_ values of the [AZ-ODC]-CCND1 and [AZ-CCND1]-ODC complexes were also determined. Figure 5 shows the size distribution plots obtained for these protein complexes at different molar ratios of AZ-ODC/CCND1 (Figure 5A) or AZ-CCND1/ODC (Figure 5B). When the molar ratio of AZ/ODC was fixed at 1, increasing CCND1 concentrations made the size distribution plot gradually shift to the right, indicating formation of the ODC-AZ-CCND1 complex. The dissociation and association of AZ-ODC and CCND1 were also quantitatively measured. The *K*_d_ of the [AZ-ODC]-CCND1 complex was approximately 4.93 μM, which was considerably larger than that of AZ_WT_-CCND1 (0.81 μM, Table 1), demonstrating that CCND1 binds to the AZ-ODC complex 6-fold more weakly than to AZ alone. The *K*_d_ of the [AZ-CCND1]-ODC complex was approximately 1.26 μM, which was also considerably larger than that of AZ-ODC (0.21 μM, Table 1), demonstrating that ODC binds to the AZ-CCND1 complex 6-fold more weakly than to AZ alone. These data indicate that although the binding sites for ODC and CCND1 in the AZ protein are different, the binding affinity of ODC (or CCND1) toward AZ is influenced by the binding of CCND1 (or ODC) to AZ.

### AZ-mediated ODC and CCND1 protein degradation

**Figure 6:**
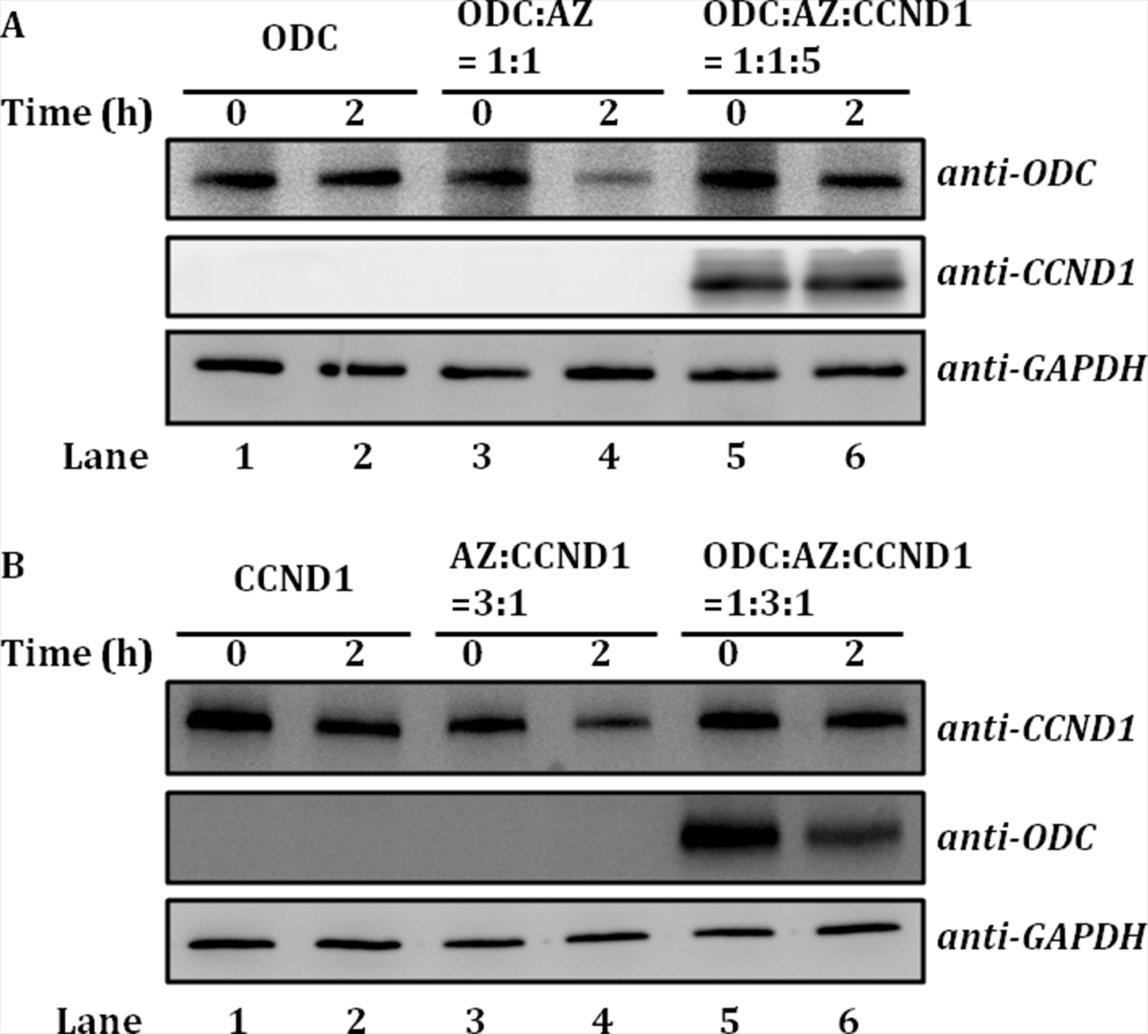
AZ-mediated ODC and CCND1 protein degradation The degradation of recombinant ODC or CCND1 protein in the presence of AZ was examined by reticulocyte lysate-based *in vitro* degradation. Human anti-ODC and anti-CCND1 antibodies were utilized as probes in subsequent immunoblotting experiments. **A.** AZ-mediated ODC degradation. Lanes 1 and 2: ODC only. Lanes 3 and 4: ODC mixed with AZ; the molar ratio of [ODC]/[AZ] was 1:1. Lanes 5 and 6: ODC-AZ complex mixed with CCND1; the molar ratio of [ODC]/[AZ]/[CCND1] was 1:1:5. **B.** AZ-mediated CCND1 degradation. Lanes 1 and 2: CCND1 only. Lanes 3 and 4: CCND1 mixed with AZ; the molar ratio of [CCND1]/[AZ] was 1:3. Lanes 5 and 6: CCND1-AZ complex mixed with ODC; the molar ratio of [ODC]/[AZ]/[CCND1] was 1:3:1.

To study whether ODC-AZ-CCND1 ternary complex formation has an effect on the degradation of ODC and CCND1, ODC and CCND1 degradation in the presence of AZ was examined by reticulocyte lysate-based *in vitro* degradation, and the results were visualized by immunoblotting using human anti-ODC and anti-CCND1 antibodies (Figure 6).

According to the sedimentation velocity experiments, ODC-AZ-CCND1 ternary complexes formed when CCND1 was in excess (Figure 5A). Therefore, we performed these assays at a 1:1:5 molar ratio of ODC/AZ/CCND1. In the absence of AZ, ODC protein was not degraded (Lanes 1 and 2, Figure 6A), whereas ODC protein was notably degraded in the presence of AZ (Lanes 3 and 4, Figure 6A). However, when CCND1 was present and the ODC-AZ-CCND1 complex was formed, AZ-mediated ODC degradation was inhibited, although CCND1 was simultaneously degraded (Lanes 5 and 6, Figure 6A). These data indicate that CCND1 interferes with AZ-mediated ODC degradation and that the formation of ternary complexes may prevent the degradation of ODC.

It has been shown that CCND1 is degraded via a ubiquitin-independent pathway through AZ binding (18). Here, AZ-mediated CCND1 degradation in the absence or presence of ODC was also examined. CCND1 protein was not degraded by the reticulocyte lysate system (Lanes 1 and 2, Figure 6B) but was markedly degraded when AZ was present in excess (Lanes 3 and 4, Figure 6B). However, when ODC was present and the ODC-AZ-CCND1 complex was formed, AZ-mediated CCND1 degradation was inhibited, although ODC was simultaneously degraded (Lanes 5 and 6, Figure 6B). These data further indicate that ODC impedes AZ-mediated CCND1 degradation and that ternary complex formation may protect CCND1 from degradation.

## DISCUSSION

### AZ binds to its interacting proteins with different binding affinities and through different domains

The AZ-interacting proteins ODC, AZI and CCND1 show different binding affinities toward AZ. Our previous work revealed that AZI exhibits a 10-fold higher binding affinity toward AZ than does ODC [50] and that these two proteins compete for the same binding site in AZ within its C-terminal domain [58]. In fact, the minimal AZ peptide from residues 95 to 176 (AZ_95–176_) was fully functional with respect to its binding to and inhibition of ODC and AZI [58]. In addition, we suggested that the AZ N-terminus up to amino acid 94 (1–94) is not required for binding to or inhibition of ODC, as the C-terminal AZ peptide (AZ_95–228_) and the full-length AZ protein inhibit ODC in a comparable manner [58].

**Figure 7:**
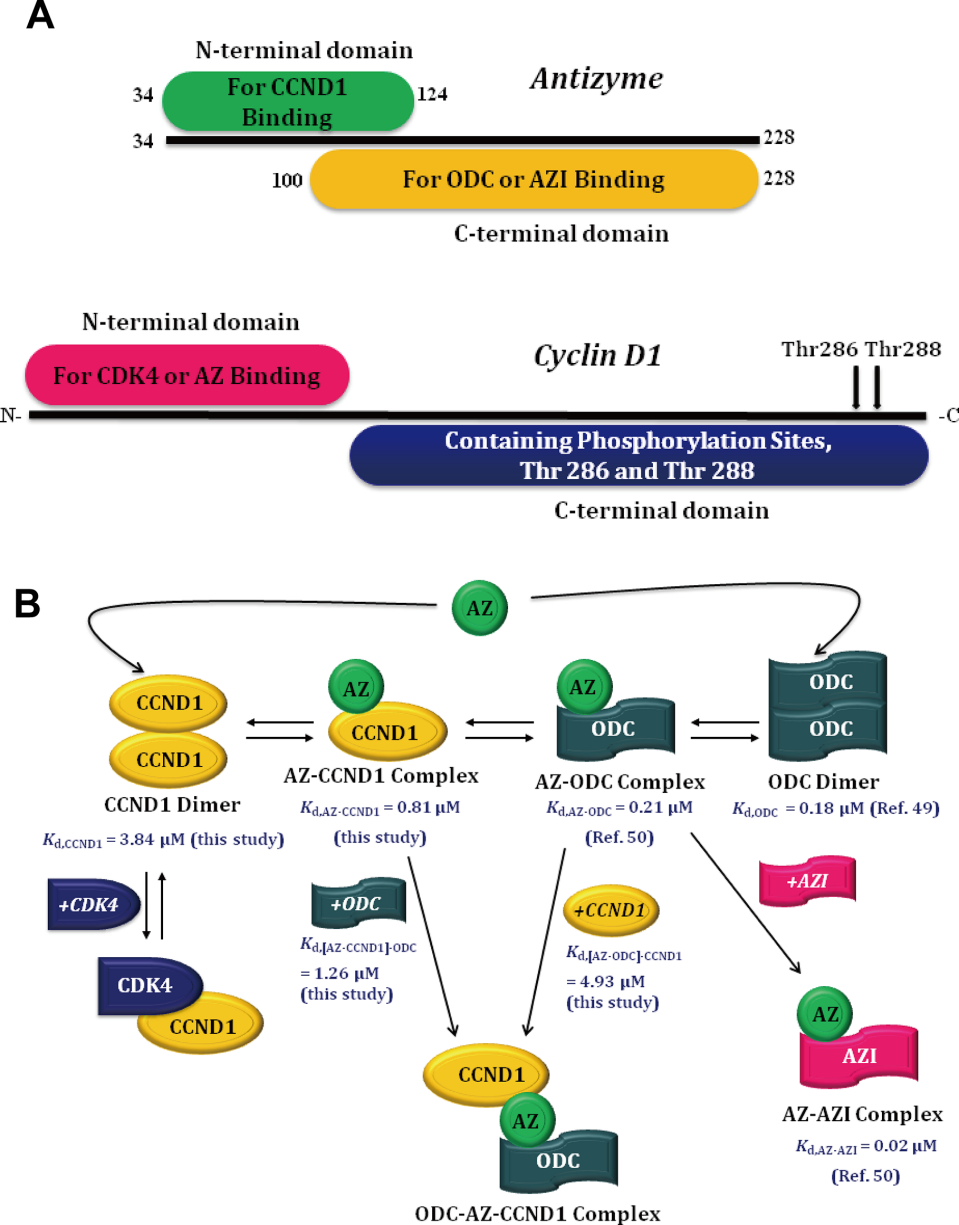
Molecular interactions between AZ and its interacting proteins **A.** Binding elements within AZ and CCND1. **B.** Mode of AZ binding to its interacting proteins.

In the present study, the AZ-binding affinity of CCND1 was determined, revealing a 4-fold weaker binding affinity toward AZ than that of ODC and an approximately 40-fold weaker binding affinity toward AZ than that of AZI (Table 1). Furthermore, the putative binding site of CCND1 may reside in the N-terminal domain of AZ, rather than in the C-terminal domain, and CCND1 binds to the N-terminal AZ peptide (AZ_34–124_) in a manner comparable to its binding to full-length AZ (Table 1). Thus, ODC (or AZI) and CCND1 occupy different regions of AZ, with the former binding to the C-terminal domain and the latter to the N-terminal domain (Figure 7A).

### Possible effect of AZ binding to CCND1

Previous studies have shown that AZ may bind to CCND1 and thereby decrease the cellular levels of CCND1 through a ubiquitin-independent pathway [19]. Our data specifically suggest that AZ can bind to the N-terminus of CCND1, rather than to the C-terminus, where the CCND1 phosphorylation sites are located (Figure 7A). Because phosphorylation at Thr 286 and/or Thr 288 at the C-terminus of CCND1 is essential for the protein to proceed through the ubiquitin-dependent degradation pathway [20–27], binding of AZ may not prohibit ubiquitin-dependent degradation of CCND1. Nevertheless, the binding of AZ to CCND1 may impede the function of CCND1, which acts as an allosteric activator of CDK4, a kinase that is essential for cell cycle transition from the G1 phase to the S phase. X-ray crystallography has demonstrated that the CDK4-binding site of CCND1 is located at the N-terminus [59], overlapping with the AZ-binding site (Figure 7A). Thus, binding of AZ to CCND1 may inhibit the activation/reactivation of CDK4 by CCND1 and thereby cause G_1_ arrest during the cell cycle [19]. Further experiments focused on the role of AZ in the ubiquitin-dependent degradation of CCND1 and on inhibition of the activation/reactivation of CDK4 by CCND1 are needed to prove these hypotheses.

### Mode of AZ binding to its interacting proteins

Based on the differential affinities determined among CCND1, ODC and AZI, a model of AZ binding to its interacting proteins is proposed here (Figure 7B). Under physiological conditions, if the cellular concentration of CCND1 is higher than that of ODC and if AZ is present, the AZ-CCND1 complex and the ODC-AZ-CCND1 ternary complex would be considerably increased in cells. Several ODC dimers might exist and produce polyamines, and the cellular concentration of CCND1-CDK4 would be suppressed. In addition, AZ-CCND1 and ODC-AZ-CCND1 complex formation would reduce the binding affinity of ODC toward AZ (*K*_d, AZ-ODC_: 0.21 μM; *K*_d, [AZ-CCND1]-ODC_: 1.26 μM), thereby inhibiting AZ-mediated ODC degradation. As a consequence, overexpression of CCND1 would be associated with ODC retention in cells.

Conversely, if the cellular concentration of ODC is higher than that of CCND1 and if AZ is present, the AZ-ODC complex would be the predominant form, accompanied by little ODC-AZ-CCND1 complex formation in cells. Under these conditions, the binding affinity of CCND1 toward AZ would be reduced (*K*_d, AZ-CCND1_: 0.81 μM; *K*_d, [AZ-ODC]-CCND1_: 4.93 μM), and the cellular concentration of CCND1-CDK4 might be elevated. Although AZ can antagonize ODC and CCND1 by binding to these proteins and can promote their degradation, if ODC were overexpressed, the cellular concentration of CCND1-CDK4 would be high, and the degradation of CCND1 would be inhibited, causing the polyamine concentration to be increased and the cells to grow continually. This situation would be worsened if AZI were simultaneously overexpressed in cells because the excess AZI would counteract the AZ-mediated degradation of ODC and CCND1. Therefore, the cellular ODC dimers and CCND1-CDK4 complex would be stable, and abnormal cell growth would be promoted.

In summary, the protein-protein interactions between AZ and its interacting proteins have been defined in this study. The *K*_d_ values for the interactions between AZ and its interacting proteins were measured precisely, and this information will greatly enhance our understanding of these interactions within the cell. However, the molecular interactions among AZ, CCND1 and CDK4 have not been investigated, so further experiments will be required to elucidate the effects of these protein interactions on the cell cycle.

## MATERIALS AND METHODS

### Expression and purification of recombinant AZ and CCND1

The human AZ and CCND1 genes were sub-cloned into the pQE30 vector (Qiagen, Hilden, Germany), which contains an N-terminal His6·Tag sequence to allow protein purification. An expression vector containing the desired gene was transformed into the JM109 strain of *Escherichia coli*, and protein expression was induced with 0.3 mM isopropyl-1-thio-β-D-galactoside (IPTG) at 25°C or 20°C. Ni-NTA Sepharose (Sigma) was then used to purify the overexpressed proteins.

The lysate-Ni-NTA mixture was first washed with a buffer containing 10 mM imidazole, 500 mM NaCl and 30 mM Tris-HCl (pH 7.6) to eliminate most of the unwanted proteins. Subsequently, the AZ or CCND1 was eluted using elution buffer containing 250 mM imidazole, 500 mM NaCl, 2 mM β-mercaptoethanol and 30 mM Tris-HCl (pH 7.6). The purified CCND1 protein was buffer exchanged and concentrated using 30 mM Tris-HCl (pH 7.6) and 2 mM β-mercaptoethanol, whereas the purified AZ protein was subjected to buffer exchange and concentration using 250 mM NaCl, 30 mM Tris-HCl (pH 7.6) and 2 mM β-mercaptoethanol. Protein purity was assessed using sodium dodecyl sulfate-polyacrylamide gel electrophoresis (SDS-PAGE), and protein concentrations were estimated using the Bradford method [60].

### Construction of truncated AZ and CCND1 mutants

Plasmids harboring a truncated human AZ or CCND1 mutant were generated via deletion mutagenesis using the QuikChange™ kit (Stratagene, La Jolla, CA, USA). The primer for the truncated mutant had to be at least 40 bases in length, with 15 bases on both sides of the deletion to complement the template DNA. For PCR amplification, purified human AZ or CCND1 DNA was used as the template, and the high-fidelity *Pfu* DNA polymerase and specific primers with the desired codons were employed to produce the specific mutated DNA sequence. The lengths of the primers designed with the preferred mutation sites were between 25 and 45 bases, which was necessary to achieve specific binding to the template DNA. Mutated plasmids with staggered nicks were generated after 16–18 amplification cycles. The wild-type human AZ or CCND1 template in the PCR products was cleaved by treatment with DpnI. The nicked DNAs with specific mutations were then used to transform the XL-10 *E. coli* strain, and the DNA sequences were confirmed by autosequencing.

### Size distribution analysis via analytical ultracentrifugation

A Beckman Optima XL-A analytical ultracentrifuge device was used to perform the sedimentation velocity experiments. Buffer (400 μl) and sample solutions (380 μl) were loaded separately into the double-sector centerpiece of a Beckman An-50 Ti rotor, and a rotor speed of 30,000 rpm was applied in the sedimentation velocity experiments. The protein samples were analyzed based on UV absorbance at 280 nm in continuous mode, with a time interval of 420 s and a step size of 0.002 cm. Numerous scans performed at different collection times were fitted to a continuous size distribution model using SEDFIT software [61, 62]. All of the size distributions were estimated at a confidence level of *p* = 0.95, a best-fit average anhydrous frictional ratio (*f/f*_0_) and a resolution (*N*) of 250 sedimentation coefficients between 0.1 and 20.0 S.

To precisely determine the *K*_d_ of CCND1 in monomer-dimer equilibrium, sedimentation velocity experiments were performed at three different protein concentrations, and all of the sedimentation data were globally fitted to the monomer-dimer equilibrium model using the program SEDPHAT [63, 64]. To determine the *K*_d_ of the AZ-CCND1 complex, sedimentation velocity experiments were performed at three different concentrations of CCND1 in the presence of a constant concentration of human AZ. Additionally, to calculate the *K*_d_ values of the heterodimers, the sedimentation data were globally fitted to the AB hetero-association model using the program SEDPHAT [63, 64]. The partial specific volumes of the proteins, the solvent densities and the viscosities were also calculated using the program SEDNTERP [65].

### Measurement of *in vitro* degradation in a reticulocyte lysate-based system

This non-radioactive detection method was slightly modified from a protocol in a previous report [66]. In particular, aliquots of purified recombinant ODC, AZ, and CCND1 were first added at various molar ratios to a 50-μl reaction containing 40 mM Tris-HCl (pH 7.5), 5 mM MgCl_2_, 2 mM DTT, 0.5 mM ATP, 10 mM phosphocreatine, 1.6 mg/ml creatine phosphokinase and 10 μl of rabbit reticulocyte lysate and were incubated for 2 hours at 37°C. To stop the reaction, loading dye was added, and the samples were boiled at 90°C for 5 minutes. The samples were then separated by SDS-PAGE and transferred to PVDF membranes for immunoblotting using anti-ODC and anti-CCND1 antibodies as probes.

## ABBREVIATION

AZ: antizyme
CCND1: cyclin D1
ODC: ornithine decarboxylase
AZI: antizyme inhibitor
AUC: analytical ultracentrifugation
